# Chemical Etching of Zinc Oxide for Thin-Film Silicon Solar Cells

**DOI:** 10.1002/cphc.201100738

**Published:** 2011-12-08

**Authors:** Jürgen Hüpkes, Jorj I Owen, Sascha E Pust, Eerke Bunte

**Affiliations:** [a] IEK5-Photovoltaik, Forschungszentrum Jülich GmbH52425 Jülich (Germany), Fax: (+49) 2461 61 3735; [b] Euroglas GmbH SOLARDammühlenweg 60, 39340 Haldensleben (Germany)

**Keywords:** electrochemistry, etching, interfaces, solar cells, zinc oxide

## Abstract

**Abstract:**

Chemical etching is widely applied to texture the surface of sputter-deposited zinc oxide for light scattering in thin-film silicon solar cells. Based on experimental findings from the literature and our own results we propose a model that explains the etching behavior of ZnO depending on the structural material properties and etching agent. All grain boundaries are prone to be etched to a certain threshold, that is defined by the deposition conditions and etching solution. Additionally, several approaches to modify the etching behavior through special preparation and etching steps are provided.

## 1. Introduction

Thin-film silicon solar cells rely on light trapping to improve their conversion efficiency from light to electrical energy. Often this is achieved by textured surfaces that scatter the light efficiently into the absorber layer. This texture has been introduced in the transparent front contact^[1]^ or in reflectors.^[2]^ Zinc oxide is one of such texturizable transparent contacts that can be prepared by sputter-deposition and subsequently textured by chemical etching on very large-area substrates.^[^3^]^ Even though the chemical etching of crystalline ZnO has been investigated in the 1960s^[^4^]^ and etching can be explained on the basis of a dangling bond model,^[^5^]^ significant complexity arises from the polycrystalline nature of sputter-deposited ZnO thin films. Since the etch rate is strongly dependent on the crystalline orientation,^[^4^,^ 6^]^ anisotropic etching occurs on structurally heterogeneous ZnO films. Etching of ZnO has been utilized for light scattering in thin-film silicon solar cells^[7]^ and therefore the need to understand the mechanism of structure formation upon etching of polycrystalline films has arisen. Several empirical studies investigated the influence of preparation and etching parameters to optimize the resulting light-scattering ZnO surface.^[8]^ However, due to the complexity of the interrelation between preparation conditions, material properties, and etching process there is neither a microscopic understanding nor any empirical model available that sufficiently describes the resulting etched structures, based on information that are accessible prior to etching.

Herein we briefly review the relevant literature and combine the available experiences and observations into an etching model. The model describes qualitatively the influence of sputter conditions, material properties, and etching conditions.

## 2. Results and Discussion

### 2.1. Empirical Growth and Etching Studies

**Figure 1:**
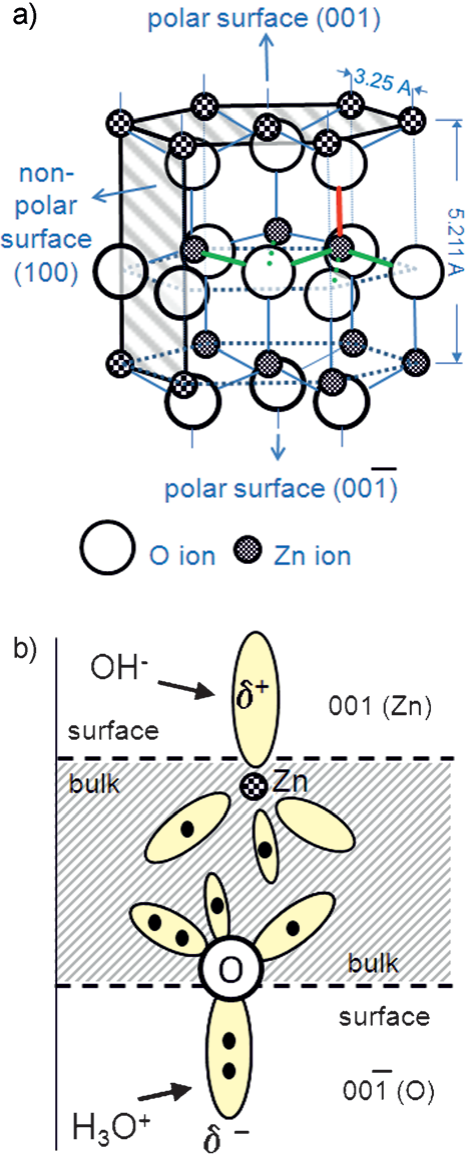
a) Wurtzite structure and b) dangling bond model for etching of a ZnO single crystal. Dotted lines are either projection lines or correspond to bonds that are hidden behind atoms.

Both ZnO single crystals and polycrystalline films are readily etched in many acidic^[^4^,^^6^^,^^9^^]^ and basic solutions.^[^4^,^^10^^]^ The etching behavior of the single crystal ZnO is well understood and can be explained by the wurtzite structure and a dangling bond model.^[^4^–^^6^^,^ 11^]^ Figure 1 shows the wurtzite structure (a) and a dangling bond model for etching at the polar surfaces (b). The surface atoms on the perfect polar faces are tightly bound to three adjacent atoms in the bulk material, while the atoms in the underlying layer are only bound to one atom in the bulk. These different bond structures between atoms inside the tightly bound double layers and from one of these double layers to the next are indicated by the green and red lines in Figure 1 a. Thus, the etch rate-determining step is to remove the tightly bound top atom. The partial positive and negative charges of the dangling bonds at the Zn (001) and O (00−1) terminated surfaces can easily be attacked by hydroxide (OH^−^) and hydronium (H_3_O^+^) ions. Due to the same charge type, the respective opposite attack is inhibited, such as H_3_O^+^ on the Zn-terminated surface. In this case, the attack of etching species can only occur at defects, such as screw dislocations, where the charge repulsion is disrupted. The model shows that the etch rates on the polar (00−1) face in acids is one order of magnitude larger than on (001) faces.^[^12^]^ Note that such truncated crystal surfaces might reconstruct and relax in real crystals to minimize their surface energy.^[^13^]^ However, most experimental findings on etching of ZnO are in agreement with the predictions of this model.

**Figure 2:**
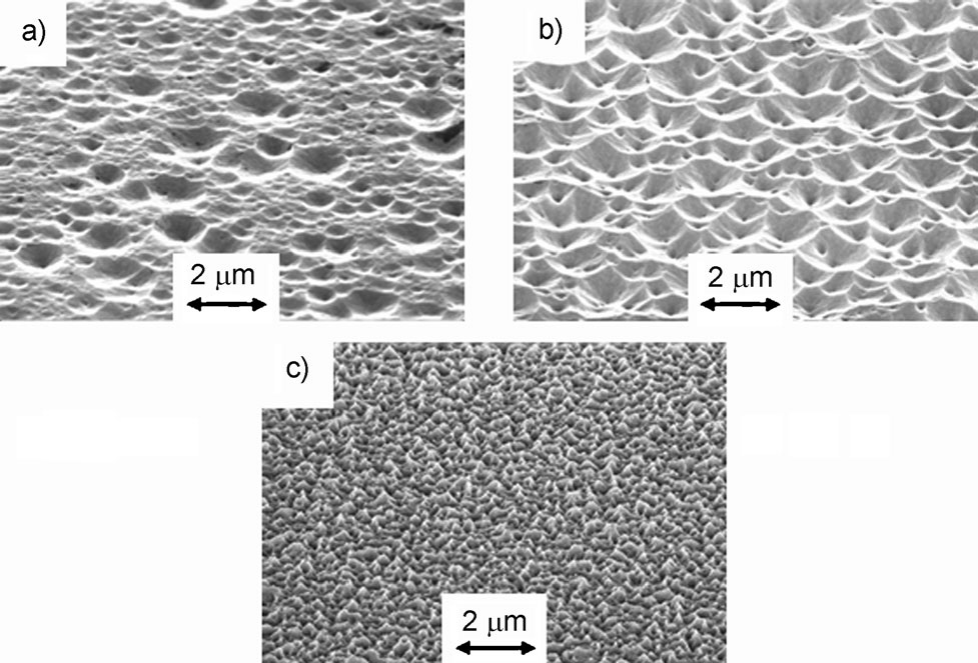
Scanning electron micrographs showing three different surface structures of polycrystalline sputtered ZnO films after etching in diluted HCl: a) low etch rate, sparse crater distribution, b) low etch rate, regular crater distribution, c) high etch rate, fine granular structure.

For polycrystalline zinc oxide the situation is much more complex. Each visible grain at the surface or in a cross section might be composed of several crystallites or contain defects.^[^14^]^ Strain is induced on the microscopic level through dopants^[15]^ or through atomic peening from the sputtering process,^[^16^]^ and macrostrain arises from the mismatch of the thermal expansion coefficient of ZnO and the substrate after high-temperature deposition.^[^17^]^ All these effects together with grain boundaries and defects inside the grains will influence the etching behavior. Several studies investigated the relationship between preparation, etching conditions and etching behavior.^[^8a^,^ c^,^ f^,^ 9a^–^d^,^ 10b^, 18]^ Some groups use the etching characteristics to determine the polarity of ZnO films by comparing etch rates and distinguishing craters from hillocks.^[^9b^]^ Others used the etching process to shape the surface in order to pattern surfaces for various device applications.^[^8a^,^ f^,^ 9d,e^, 19]^ Generally, it is observed that the etch rate varies with pH by orders of magnitude and that zinc oxide is only stable in a narrow window of pH values.^[^20^]^ Additionally, the etch rate strongly depends on the preparation of ZnO^[^8a^,^ 9a,b^]^ or on post-treatment conditions of ZnO.^[^8 g^,^ 21^]^ Further, different surface features, ranging from craters to hillocks or granular features, develop on materials with low or high etch rate.^[^8a,b^,^ 9a^]^ Exemplarily, three different surface structures of polycrystalline sputtered ZnO films are presented in Figure 2 after etching in diluted HCl. In contrast to Hickernell,^[^9a^]^ we found that the defect density, seen as crater density, can be modified over a wide range through variation of the deposition conditions applied.^[^10b^]^

Kluth et al.^[^8a^]^ proposed a phenomenological structure zone model for the etching, based on the growth models of Movchan and Demchishin^[^22^]^ as well as Thornton,^[^23^]^ that relates the sputter pressure and substrate temperature to the film structure and the resulting etching characteristics. The characteristic parameter to describe the ZnO material is the compactness, with more compact ZnO films exhibiting lower etch rates. Highly compact ZnO films develop craters upon etching with low (Figure 2 a) or medium crater density (Figure 2 b), and low compactness leads to small etching structures (Figure 2 c). The model was extended to describe the influence of the oxygen content in the sputtering atmosphere and the aluminum doping concentration in the target.^[^8a^,^ c^,^ 10b^]^ Additionally, compactness increases with an increase in film thickness^[^14^]^ and high deposition rates,^[^9a^,^ 24^]^ as well as post-deposition treatments.^[^8g^,^ 21^,^ 25^]^ Apart from the deposition or post-treatment conditions the glass type and barrier layers also play an important role in growth and etching behavior.^[^9a^,^^26^^]^

**Figure 3:**
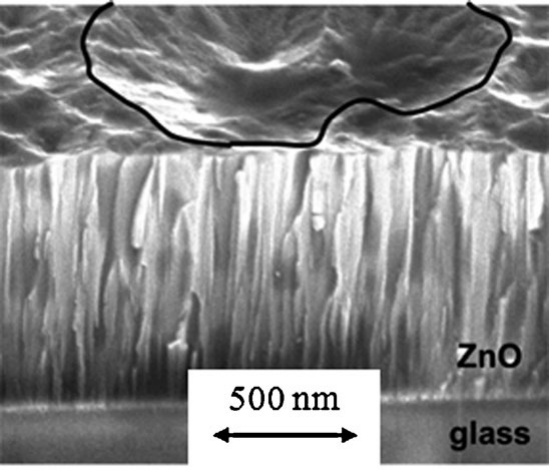
Cross section of a ZnO:Al sample imaged at an inclination angle by scanning electron microscopy to show the relationship between the columnar crystallites and an etching crater. The rim of the etching crater is marked (—).

In compact films craters form only at sparsely distributed locations and crater walls span many grain boundaries. Figure 3 shows the surface and cross section of a polycrystalline ZnO film after etching in diluted HCl. The large crater on the surface appears to span many grains. The crater rim is indicated by a solid line. From this one can conclude that typical grain boundaries do not act as a center for crater nucleation in compact ZnO films. Typically compact ZnO exhibits a strong texture with (001) direction along the substrate normal, while the observation of other orientations correlates with high etch rates and small features.^[^8b^]^ Even though the compactness cannot be directly measured before etching, some approaches correlated the etching behavior to ZnO-deposited properties, such as film structure measured by X-ray diffraction^[^1a^,^ 8c^]^ or microscopy of the deposited ZnO surface.^[^8e^,^ 14^,^ 27^]^

**Figure 4:**
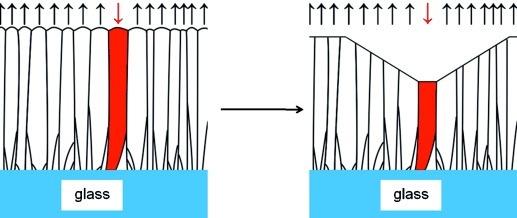
Polarity inverted model: craters form at quickly etched O-terminated grains, the crater shape is determined through the relation between vertical and lateral etch rates.

On glass ZnO films tend to form columnar grains with the c-axis normal to the surface and Zn termination, especially for highly conductive films.^[^14^,^ 28^]^ Some groups explain the different etch rates with the different polarities of the crystallites.^[^9a^]^ One model to explain the nucleation of craters is given by Szyszka et al.^[^28b^]^ The previously mentioned compact films are generally Zn-terminated. However, according to the model, some of the grains grow with opposite polarity. The sputter-deposition conditions determine the polarity of ZnO. This is illustrated in Figure 4. According to the dangling bond model high etch rates are achieved for O-terminated surfaces. Thus, deep craters are etched at those sites. The crater shape is defined by the etch rates along the different crystal faces. Due to the missing in-plane texture, there is no or only limited symmetry of the craters in polycrystalline ZnO. This model also fits the X-ray photoelectron-spectroscopy observations of Klein and Säuberlich,^[^13^]^ who concluded a tendency of the average surface termination to change with deposition conditions.

**Figure 5:**
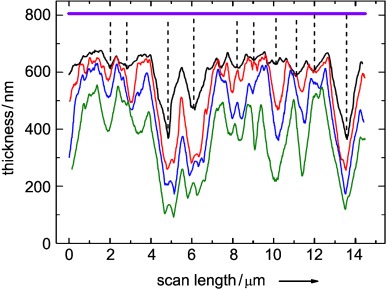
Atomic force microscopic surface profiles at approximately the same location on a ZnO:Al film after 400 s of etching in KOH (—) followed by 5 (—), 10 (—), and 20 (—) cumulative seconds in HCl. The original film thickness is indicated by a strait bold line (—).

Crater formation with different etching processes was investigated by several groups.^[^8f^,^ 10b^, 29]^ There is an important difference between acidic and basic etching for ZnO single crystals. However, in the case of polycrystalline films the density of etching features is similar to that for acidic and basic etchings, as well as after physical sputtering.^[^10b^]^ Owen et al.^[^30^]^ demonstrated that etching features, initially created in KOH, progress upon subsequent acidic etching steps in HCl. Figure 5 illustrates these observations by the thickness profiles of a single ZnO:Al film at the same position after several etching steps at room temperature. First the film was etched in 30 w/w % KOH for 400 s (black line) followed by an etching in 0.5 w/w % HCl for cumulative times of 5, 10, and 20 s (colored lines). The initial film thickness is indicated by a straight, bold line. Etching sites are marked with dashed vertical lines. HCl craters progress at the same etching sites, where they have started during the KOH etching.

These experimental results contradict the polarity model for etching. It also is unlikely that the grain orientation or polarity changes upon a moderate post-treatment,^8^ ^g, 21^ especially if only the surface is treated by an ion beam.^[^25^]^ Furthermore, the growth rates of O- and Zn-terminated grains differ by about 30 %^[31]^ and O-terminated grains would be quickly overtaken by Zn-terminations during the “survival of the fastest” growth.^[^32^]^ Thus, after this growth period, no O-terminated grains survive and no craters would be formed upon etching.^[^31b^]^

**Figure 6:**
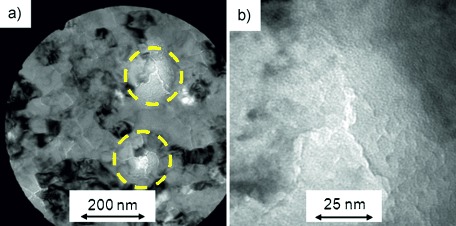
TEM images of a slightly etched ZnO:Al surface. a) Grain boundaries are visible at the center of crater formation (marked by dashed circles). b) Magnified area of one crater center.

We conclude that etching sites are given by the material and are related to the structural disorder in the ZnO:Al films and not to the orientation of certain grains. To identify the kind of disorder we performed transmission electron microscopy (TEM) investigations.^[^14^]^ TEM samples were prepared from a ZnO:Al film after initial crater formation in HCl to observe top-view images at crater locations. Figure 6 shows these areas in different magnifications. The grey-scale contrast depends on the grain orientation relative to the incident electron beam. The extended bright areas correspond to crater centers, where the specimen is thinner than the surrounding area. The bright lines between areas of different contrast are grain boundaries that always meet at crater centers. However, lots of separate grains and grain boundaries are distributed on the whole image, which do not act as etching sites.

**Figure 7:**
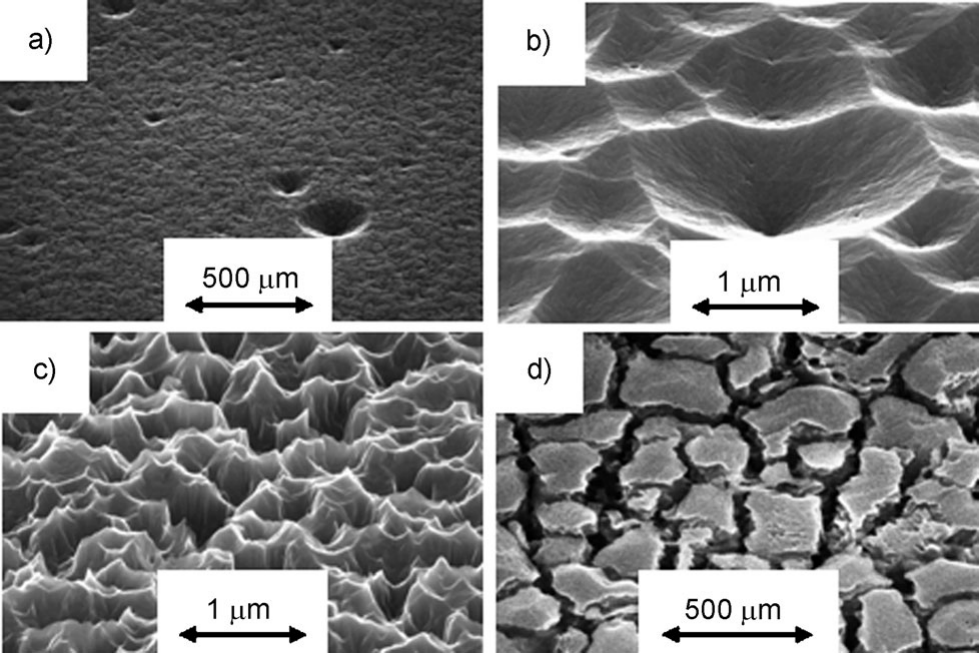
Scanning electron micrographs of accessible etched morphologies after a single etching step of identical polycrystalline ZnO:Al material: a) waterless etching in a 0.5 w/w % solution of HCl in 1,4-dioxane, b) etching in a 0.5 w/w % aqueous solution of HCl (as an example for protic acids with a high degree of dissociation), c) etching in a 1 w/w % aqueous solution of HF, d) anodic electrochemical etching in 0.1 m KCl at +2 V vs. Ag|AgCl|3 m KCl.

Figure 7 gives an overview on the topographies accessible on the “standard Jülich” ZnO:Al^[^8c^]^ through the different etching methods, which are

1. waterless etching without dissociation of the involved acid (Figure 7 a),
2. etching in aqueous solutions of HCl or other protic acids with a high degree of dissociation (Figure 7 b),^[^8c^]^
3. etching in HF (Figure 7 c),^[^29d^]^ and
4. anodic electrochemical etching (Figure 7 d).^[^8 h^,^ 33^]^

The fundamental difference between these processes is the density of points-of-attack. While under waterless etching conditions only a few grain boundaries are attacked by the etchant, the electrochemical etching procedure selectively removes all grain boundaries. The HCl- and HF-based processes place themselves in between these two extremes. The same trend has also been observed for ZnO thin films that were sputtered at conditions different to those of the “standard Jülich” ZnO:Al and it has been found to be valid even for ZnO single-crystal etching.^[^29d^]^ This overview shows nicely that the density of points-of-attack can be a function of the etchant, and is not solely defined by the material. In the following we go into the details of the etching to understand the different nature of the etchants.

By performing the waterless HCl etching in an inert gas atmosphere, we were able to inhibit the dissociation of HCl almost completely. The inhibited dissociation resulted in a significantly lower amount of attack points (Figure 7 a) as compared to the standard aqueous HCl etching (Figure 7 b). In addition to these few big craters a homogeneous removal of the material was observed, ascribed to a thermodynamically limited etching behavior with an integral etch rate of approximately 1.6 nm s^−1^. A very simplified explanation for this behavior is the presence of mostly undissociated HCl molecules at the solid/liquid interface. Due to the lack of water, only a very small amount of these molecules, striving for thermodynamical equilibrium, dissociates and supplies a few protons very close to the interface or in vicinity of etching-sensitive grain boundaries. These protons are able to initiate etching events. However, crater growth is then inhibited by the fact that not enough protons are available to lead to a continuous etching. With the exposition of ZnO to a water based acid (Figure 7 b and Figure 7 c) acidic species are statistically distributed at the ZnO surface. As a result, etching could occur anywhere at the interface. According to the etching of ZnO single crystals in acid craters develop at certain grain boundaries. The density of craters is then determined by the material properties, type of the etchant, and etching conditions.^[^14^,^ 29d^]^ Upon utilizing an anodic electrochemical process in an electrolyte solution, however, the interfacial reaction is limited to the grain boundaries (Figure 7 d), leaving the ZnO columns almost unaffected.^[^8 h^,^ 33^]^ This is due to the fact that in the course of this anodization process, protons are electrochemically generated exclusively at the grain boundaries and immediately consumed at the ZnO thin film, leading to a locally limited dissolution of the ZnO. The process itself also provides strong evidence for the grain boundaries being the source of any etching event, as it has been described before on the basis of TEM experiments.^[^14^]^

## 2.2 Polycrystalline ZnO:Al Etching Model

The observations of the previous sections are used to formulate three postulates for a polycrystalline ZnO etching model.

**Figure 8:**
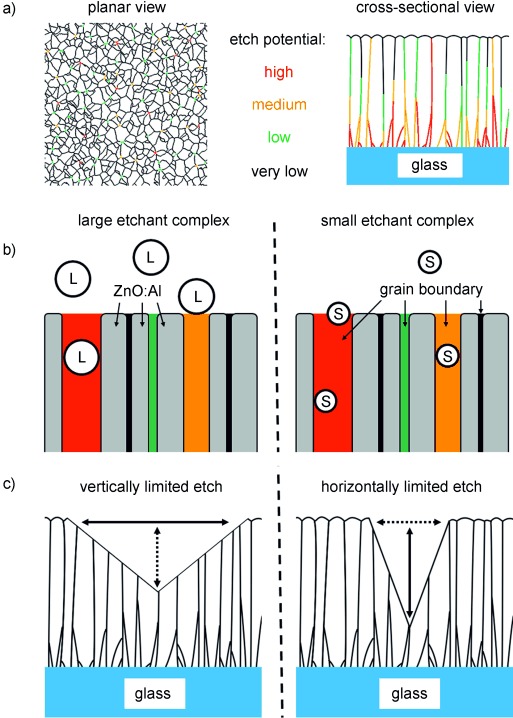
Diagrams of the three postulates of the etching model: a) planar (left) and cross-sectional (right) views of a ZnO film with grain boundaries of different etch potentials, b) interaction of the etchants with the ZnO film depending on the etchant size: large (left) and small etchant (right), at grain boundaries of different etch potentials, c) resulting crater shape for large and small etchants with vertically (left) and laterally (right) limited etch rates.

1. Sputtered ZnO:Al is grown Zn-terminated. This (001) plane at the film surface, similar to Zn-terminated single crystals, nearly inhibits the etching of the ZnO grains, however, every grain boundary has a certain potential to be etched. This etch potential depends on the compactness of the grain boundary, less compact (more porous) regions having a higher potential for etching. Figure 8 agives a schematic representation of this postulate, indicating the variation in grain boundary etch potentials by different colors for the grain boundaries. The etch potential varies not only across the surface (Figure 8 a, left), but also perpetuates vertically through the film (Figure 8 a, right). Craters are formed at grain boundaries with less order, having higher potentials for etching than those with more order. A physical explanation for this behavior is that less ordered grain boundaries can be more easily accessed by the etching agent, allowing etching on a primarily Zn-terminated polycrystalline ZnO surface as at grain boundaries other planes are accessible to the etchant. The primarily Zn-terminated polycrystalline ZnO surface can be etched only if the etching agent can access a disordered grain boundary to attack other crystal planes.

It was observed that stopping and restarting the etching process does not affect the etching sites, etching returns every time to the same positions, and that basic and acidic solutions etch at the same locations.^[^30^]^ From these observations it can be concluded that the sites of accelerated etching are built into the films during deposition rather than catalysis through surface adsorbates. A physical reason for this etching behavior is related to the columnar growth of sputtered ZnO:Al. After the nucleation and “survival of the fastest” growth periods, the column size remains fairly constant (Figure 3), and grain boundaries are simply perpetuated with increased thickness as further growth does not create more nuclei, but thickens the previously grown crystals. The etch potential of the grain boundaries, however, can change during further growth and typically is reduced with film thickness (Figure 8 a, right).

1. The etchant solution and conditions define an etching threshold. Grain boundaries with etch potentials above the threshold are more actively etched. This threshold depends on the size and the concentration of the etchant complex involved. Figure 8 b gives a schematic representation of this postulate.

It was observed that the density of craters increases with increasing HCl temperature.^[^14^]^ Thus, an increasing temperature lowers the threshold, allowing more grain boundaries with lower etch potentials to be etched, as confirmed by single crystal ZnO etching.^[^14^]^ A possible physical explanation considers the size of the ionic clusters formed by the hydronium ion together with neutral water molecules (H_5_O_2_^+^, H_9_O_4_^+^ or even stronger hydrated species). At lower temperatures large clusters are formed with the surrounding polar water molecules in order to delocalize the positive charge.^[34]^ As the temperature of the solution is increased the weak hydrogen bonds are broken, thereby reducing the size of the cluster ion. The smaller water hydronium clusters are then able to penetrate smaller and thus more grain boundaries compared to the larger water hydronium clusters, leading to a higher density of craters (Figure 8 b).

Similarly, it has been observed that the density of craters increases with a decrease in HCl concentration.^[^14^]^ Thus, decreasing the concentration lowers the threshold, allowing more grain boundaries with lower etch potentials to be etched as confirmed by single crystal ZnO etching.^[^14^]^ A possible physical explanation relates to the self-limiting nature of what would be the most actively etched regions in a higher concentrated solution. Due to the extremely low hydronium concentration, only a very small pH gradient is formed upon the consumption of an ion. Thus the ion is not readily replenished by another which would continue the accelerated etching. Instead, the etching progress to form large craters is prohibited and slowly etching craters are therefore favored.

HF has been shown to create a higher density of craters than HCl or other acids.^[^29d^]^ Like the temperature and concentration dependence in HCl etching, single crystal ZnO etching showed that the higher density of craters arises from the more homogeneous etching of defects.^[^29d^]^ Since dissociated HCl and HF molecules will both form large hydrated clusters, the physical explanation does not solely depend on the size of the etching agent, but also on the tendency of these molecules to dissociate. HCl and HF have acidic dissociation constants of ≪0 (total dissociation) and 3.2, respectively.^[^35^]^ Thus, in an HCl solution practically all of the dissolved HCl molecules form large water hydronium clusters. On the other hand, in an HF solution many of the molecules are not dissociated at a given time and remain relatively small compared to the water hydronium clusters. These small molecules can penetrate defects with even lower etch potential. If the HF-based solvated compound then dissociates, it can form a crater at a grain boundary, where the penetration of hydrated acidic clusters is sterically prohibited.

The fact that the unique etching characteristics stem from both the small and weak nature of the HF-based compounds has also been seen in the etching characteristics of CH_3_CO_2_H.^[^14^]^ Similar to HF this weak acid does not fully dissociate, but due to the large size of the molecule smaller grain boundaries cannot be penetrated before dissociation. Like in the case of HCl etching it is limited to the grain boundaries with large etch potentials.

It should be mentioned that this is a very simplified view of the chemical interactions taking place, and the true nature of HF in solution remains a strongly debated topic.^[^36^]^ The dissociation constant of HF is anomalously small. Giguère and Turrell suggested that HF may actually dissociate strongly, however, the interaction with water is so strong that the hydronium and fluoride ion are bound as a contact ion pair, effectively rendering HF into a weak acid.^[^37^]^ Others say that HF exists in water simply as a solvated molecule.^[^38^]^ Independent on what is the case, the HF-based compounds formed may be effectively smaller than the hydrated acidic clusters of other acids.

1. The vertical and horizontal etch rates are also defined by the solution. The vertical etch rate progresses along peculiar grain boundaries (peculiar as most of the grain boundaries do not exhibit the same etching behavior). It is limited by the nature of the grain boundary, as well as the mobility and size of the etching agents. The horizontal etch rate is limited by the concentration of etchants and the crystal structure. If the vertical etch rate is the limiting etch rate (Figure 8 c, left) the characteristic crater-opening angles formed are defined by the (101) plane. On the other hand, if the horizontal etch rate is the limiting factor and the vertical etch rate is fast due to the combination of a high etch potential of the grain boundary and the low threshold of the etchant, steeper opening angles are observed (Figure 8 c, right). During the evolution of craters with vertically limited etch rate (e.g. when etching with acids other than HF), the appearance of a dominant angle of inclination was observed. This characteristic opening angle is assumed to be related to a preferential etching on the (101) plane.^[^4^,^ 6^,^ 39^]^ For such surfaces the etching process is limited vertically (Figure 8 c, left). A physical reason for this etching behavior is similar to the one discussed in postulate (2) and relates to the size of the etching agent. In the vertically limited case large etchants cannot penetrate deeply into the ZnO:Al film and reactions are generally limited to the surface. Thus, when etching occurs at particularly large grain boundaries the etching will expand horizontally very quickly until the (101) planes are reached. Etching in HF, on the other hand, does not exhibit a characteristic opening angle, and sharper features are observed.^[^29d^]^ In this case the etching process is limited horizontally (Figure 8 c). The reactions are not limited to the surface as the small HF-based compounds can penetrate the ZnO:Al film before dissociating. Many of the HF-based species in the etchant solution are not dissociated, therefore the horizontal etch rate along the (101) plane is limited. For basic etching the etch mechanism is supposed to be similar. Depending on the etching conditions and material properties the horizontal etch rates can be very low or high, as etchants can produce steep, even up to vertical structures, or quite shallow craters into polycrystalline ZnO films.^[^39^]^

Using this etching model we designed and successfully demonstrated a new etching process for creating a regular crater distribution on very compact ZnO films prepared during industrially relevant processes.^[^8d^,^ 40^]^ In a first HF-etching step many sharp craters were created, thereby increasing the etch potential of the grain boundaries. A subsequent HCl-etching step was used to gradually widen the craters and increasing the opening angles. This method creates regular features that are well designed for solar cell applications.

## 3. Conclusions

This paper provides a literature review on wet etching of zinc oxide in acidic or basic solutions which serves as a background for the discussion of etching models. Recently, we performed some additional experiments to test previous assumptions or to clarify open questions regarding crater formation on polycrystalline films and single crystals of ZnO. Based on these experimental findings we proposed a three postulate etching model that allows us to qualitatively describe the etching behavior of ZnO thin films through the combination of physical and chemical aspects. Deposition conditions of the ZnO films determine the material properties that are modeled by mainly Zn-terminated grains, which are surrounded by grain boundaries of different etch potentials. These potentials depend on the degree of disorder between adjacent grains. The etching agent then defines a threshold by its effective size and mobility. If the etch potential of the grain boundary exceeds this threshold vertical etching along the grain boundary occurs and craters are formed. Lateral etching is related to the wurtzite structure of ZnO, and its relation to the vertical etch rate defines the shape of craters.

The aim for optimization is to carefully control the boundary threshold distribution of the grains through preparation conditions or post-treatments, and selection of etchant. This was successfully demonstrated by a two-step etching of very compact ZnO films, first in HF and then in HCl, to create regular features that are well designed for solar cell applications. To a certain extent, our model allows us to semi-quantitatively predict the morphology resulting from the etching process. It is therefore a strong contribution to the tuning of surface features for light-trapping issues on sputtered ZnO thin films.

## Experimental Section

ZnO single crystal wafers were purchased from Crystec, Berlin, Germany. The crystal wafers were cleaved from a hydrothermally grown ZnO crystal and polished on both (001) and (00−1) faces perpendicular to the c-axis. The (001) face was marked by a beveled edge. Doped (Al or Ga) or undoped ZnO films were prepared by sputter-deposition using different processes and deposition systems on various glass substrates. Most films were prepared in a vertical in-line sputtering system for 40×40 cm^2^ substrates, supplied by VAAT, Dresden, Germany.^[^10b^]^ As an example, typical sputtering conditions are given for the “standard Jülich” ZnO:Al, that is sputtered from a planar 750×100 mm^2^ ceramic ZnO:Al_2_O_3_ (99:1 w/w %) target at a substrate temperature of 300 °C and a pressure of 0.1 Pa pure argon onto a Corning Eagle XG 1.1 mm glass substrate. The plasma was excited by a radio frequency of 13.56 MHz and a power density of 2 W cm^−2^. However, some ZnO:Al films were also reactively sputtered from metallic targets. More details on the specific processes are given in the references ^[^8a^,^ c^,^ g^,^ 9a^,^ 10b^,^ 21^,^ 24^,^ 25^]^.

The standard chemical etching was performed in a 0.5 w/w % aqueous hydrochloric acid (HCl) solution at room temperature. After the wanted time of etching had elapsed, the sample was thoroughly rinsed with deionized water and dried with a nitrogen gun. Other etching procedures included etching in 1 w/w % hydrofluoric acid (HF), 30 w/w % potassium hydroxide (KOH), waterless etching, and electrochemical etching (cf. ref. ^[^8h^]^). In the case of waterless etching, ZnO films were etched in diluted HCl (0.5 w/w % in the case presented herein) with the solvent being dry 1,4-dioxane (Fluka, Munich, Germany). The experiment was performed in an inert gas atmosphere under exclusion of water. The ZnO material was characterized mainly through scanning electron, transmission electron or atomic force microscopy.
